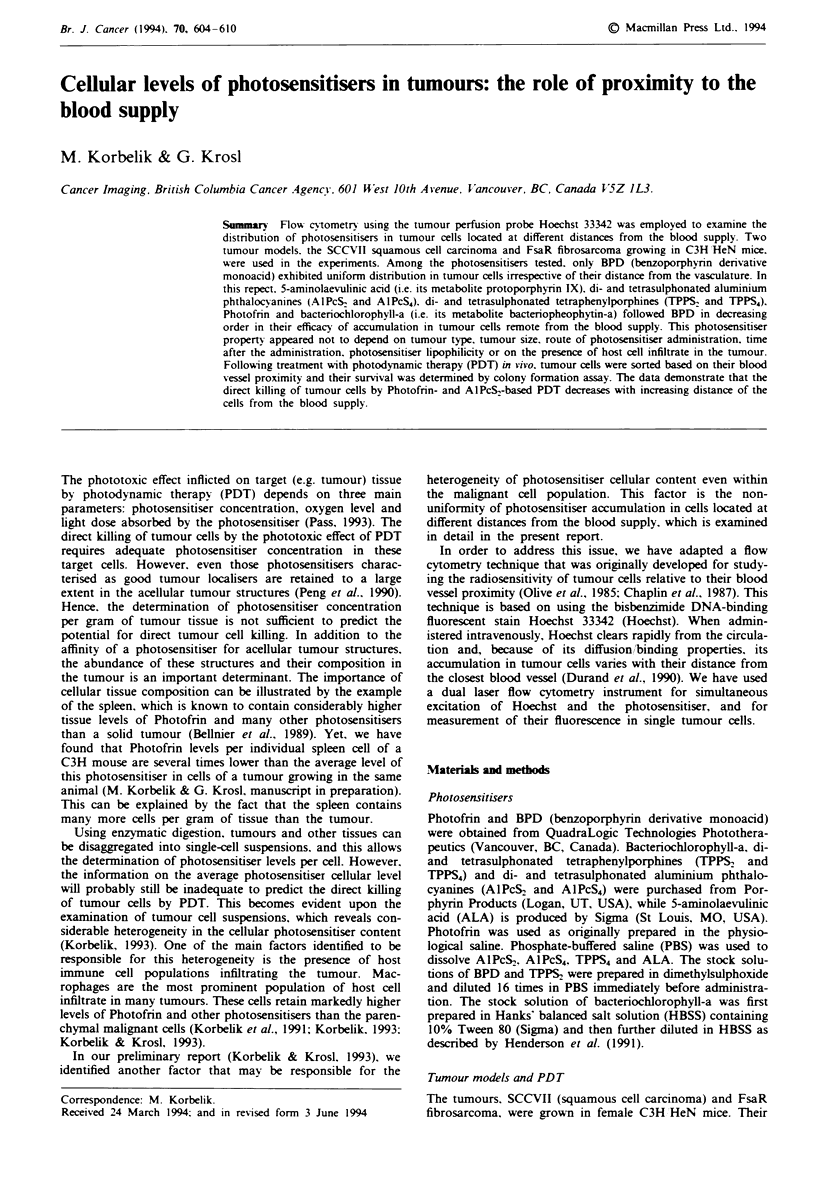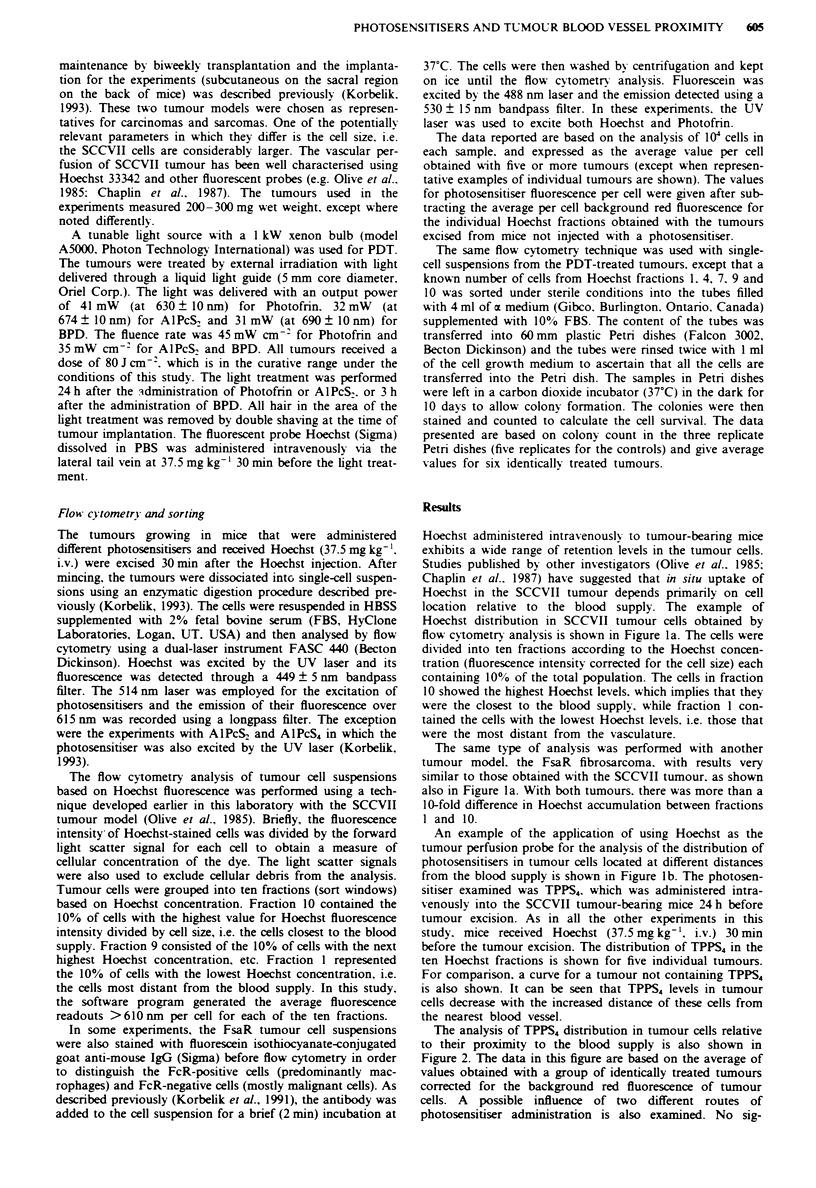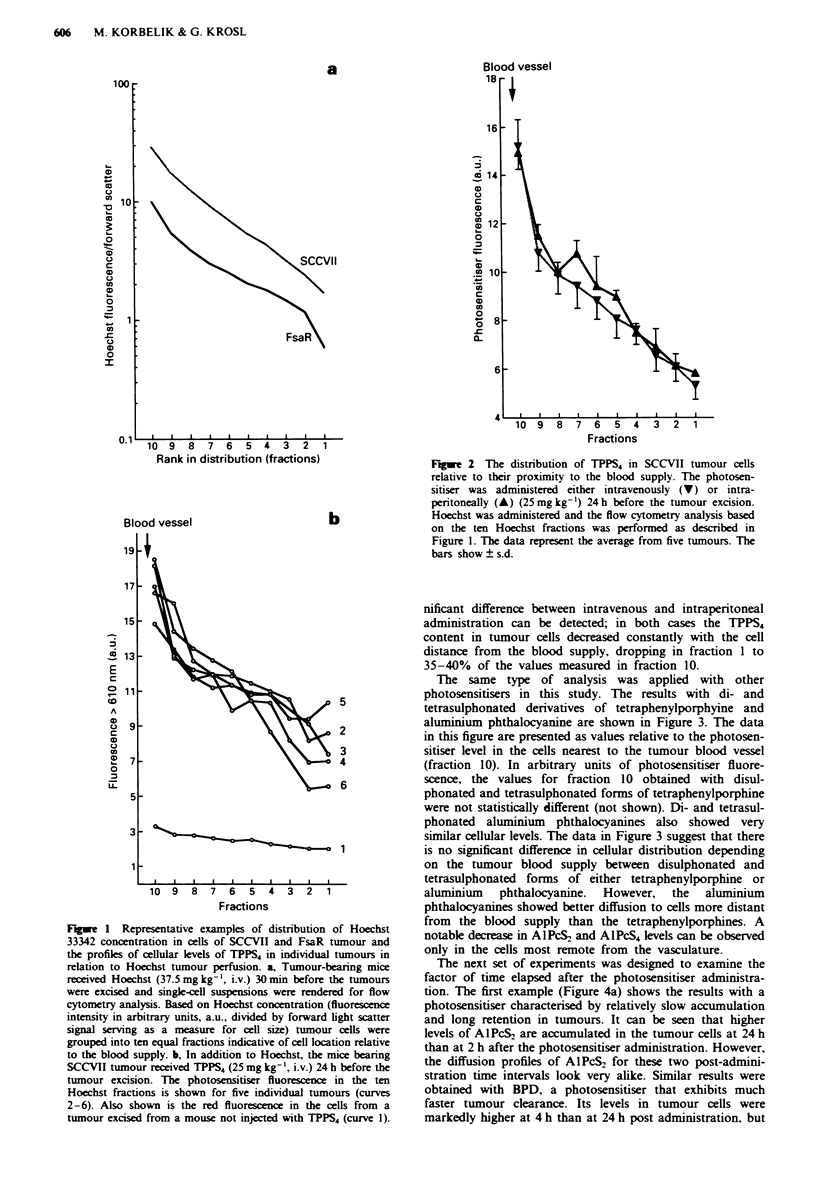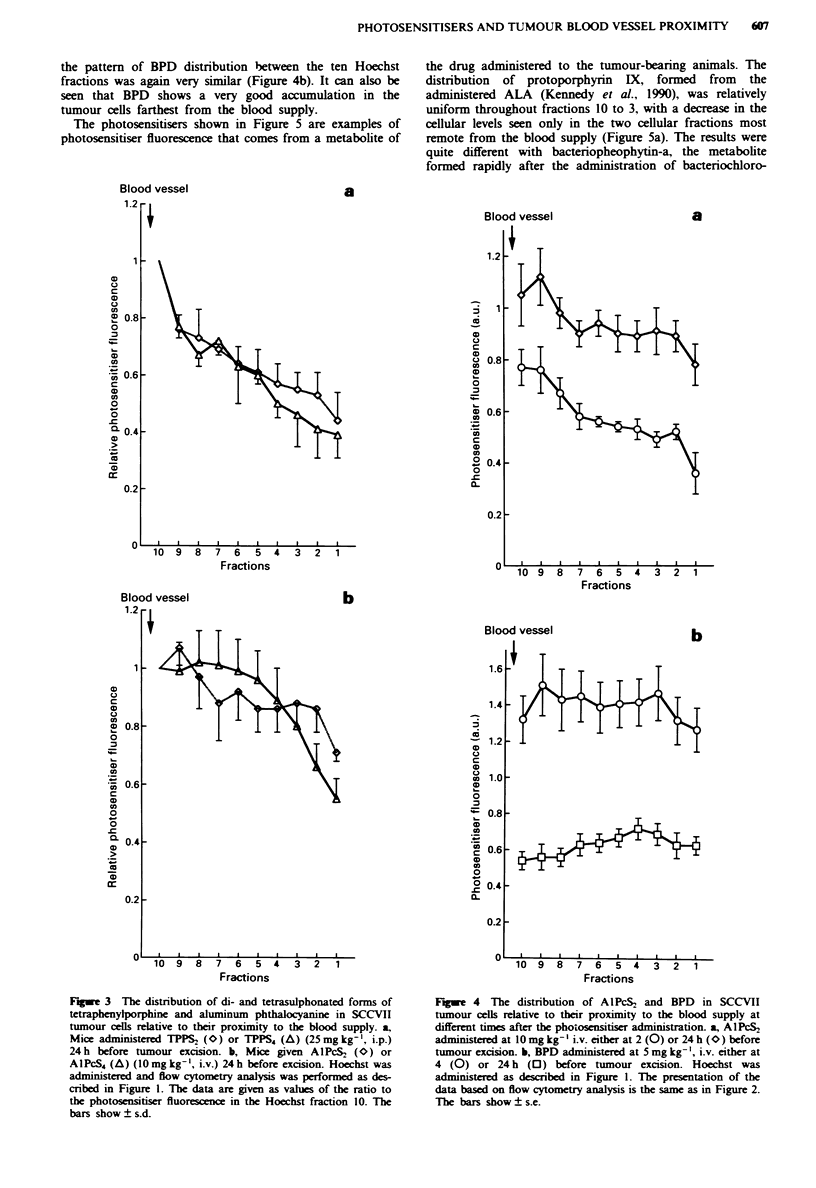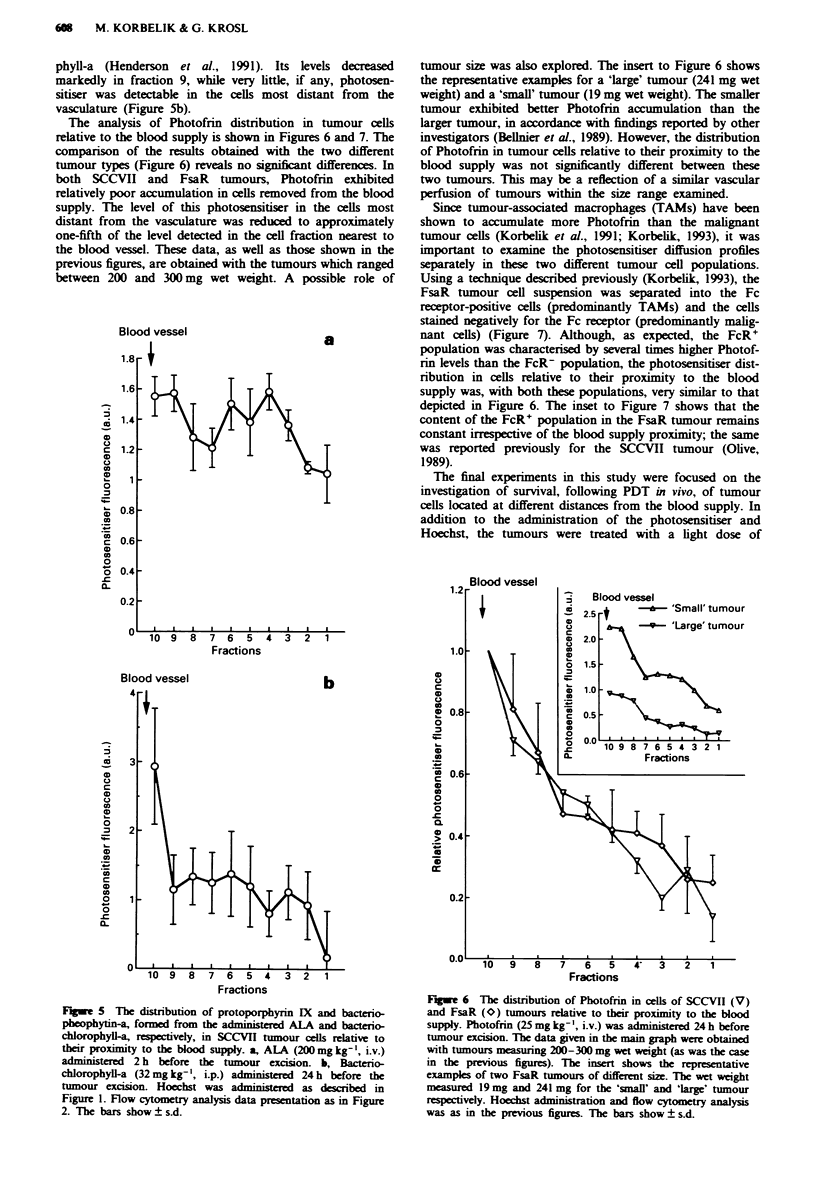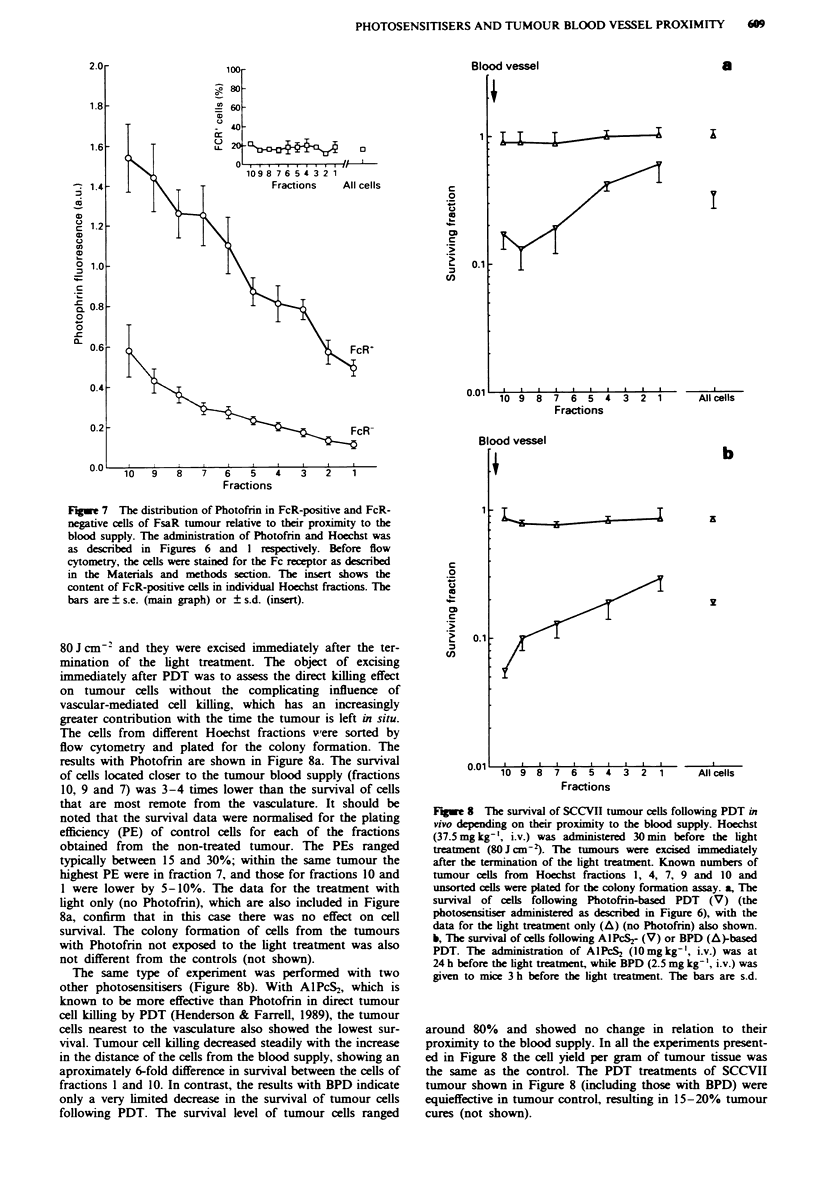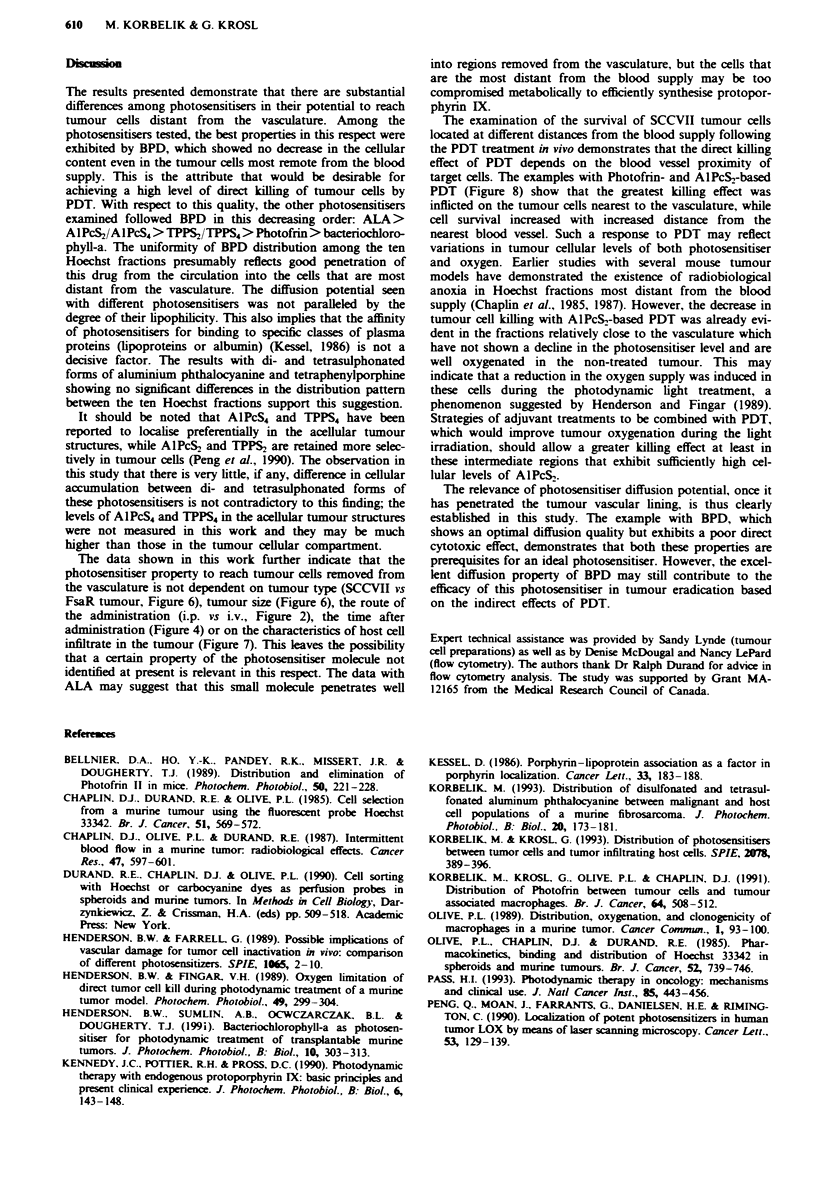# Cellular levels of photosensitisers in tumours: the role of proximity to the blood supply.

**DOI:** 10.1038/bjc.1994.358

**Published:** 1994-10

**Authors:** M. Korbelik, G. Krosl

**Affiliations:** British Columbia Cancer Agency, Vancouver, Canada.

## Abstract

Flow cytometry using the tumour perfusion probe Hoechst 33342 was employed to examine the distribution of photosensitisers in tumour cells located at different distances from the blood supply. Two tumour models, the SCCVII squamous cell carcinoma and FsaR fibrosarcoma growing in C3H/HeN mice, were used in the experiments. Among the photosensitisers tested, only BPD (benzoporphyrin derivative monoacid) exhibited uniform distribution in tumour cells irrespective of their distance from the vasculature. In this respect, 5-aminolaevulinic acid (i.e. its metabolite protoporphyrin IX), di- and tetrasulphonated aluminium phthalocyanines (A1PcS2 and AlPcS4), di- and tetrasulphonated tetraphenylporphines (TPPS2 and TPPS4), Photofrin and bacteriochlorophyll-a (i.e. its metabolite bacteriopheophytin-a) followed BPD in decreasing order in their efficacy of accumulation in tumour cells remote from the blood supply. This photosensitiser property appeared not to depend on tumour type, tumour size, route of photosensitiser administration, time after the administration, photosensitiser lipophilicity or on the presence of host cell infiltrate in the tumour. Following treatment with photodynamic therapy (PDT) in vivo, tumour cells were sorted based on their blood vessel proximity and their survival was determined by colony formation assay. The data demonstrate that the direct killing of tumour cells by Photofrin- and A1PcS2-based PDT decreases with increasing distance of the cells from the blood supply.


*Br. J. Cancer* (1994), **70**, 604-610 © Macmillan Press Ltd., 1994

### Cellular levels of photosensitisers in tumours: the role of proximity to the blood supply

M. Korbelik & G. Krosl

*Cancer Imaging, British Columbia Cancer Agency, 601 West 10th Avenue, Vancouver, BC, Canada V5Z 1L3.*

**Summary** Flow cytometry using the tumour perfusion probe Hoechst 33342 was employed to examine the distribution of photosensitisers in tumour cells located at different distances from the blood supply. Two tumour models, the SCCVII squamous cell carcinoma and FsaR fibrosarcoma growing in C3H/HeN mice, were used in the experiments. Among the photosensitisers tested, only BPD (benzoporphyrin derivative monoacid) exhibited uniform distribution in tumour cells irrespective of their distance from the vasculature. In this repect, 5-aminolaevulinic acid (i.e. its metabolite protoporphyrin IX), di- and tetrasulphonated aluminium phthalocyanines ($AlPcS_2$ and $AlPcS_4$), di- and tetrasulphonated tetraphenylporphines ($TPPS_2$ and $TPPS_4$), Photofrin and bacteriochlorophyll-a (i.e. its metabolite bacteriopheophytin-a) followed BPD in decreasing order in their efficacy of accumulation in tumour cells remote from the blood supply. This photosensitiser property appeared not to depend on tumour type, tumour size, route of photosensitiser administration, time after the administration, photosensitiser lipophilicity or on the presence of host cell infiltrate in the tumour. Following treatment with photodynamic therapy (PDT) *in vivo*, tumour cells were sorted based on their blood vessel proximity and their survival was determined by colony formation assay. The data demonstrate that the direct killing of tumour cells by Photofrin- and $AlPcS_2$-based PDT decreases with increasing distance of the cells from the blood supply.

The phototoxic effect inflicted on target (e.g. tumour) tissue by photodynamic therapy (PDT) depends on three main parameters: photosensitiser concentration, oxygen level and light dose absorbed by the photosensitiser (Pass, 1993). The direct killing of tumour cells by the phototoxic effect of PDT requires adequate photosensitiser concentration in these target cells. However, even those photosensitisers characterised as good tumour localisers are retained to a large extent in the acellular tumour structures (Peng *et al.*, 1990). Hence, the determination of photosensitiser concentration per gram of tumour tissue is not sufficient to predict the potential for direct tumour cell killing. In addition to the affinity of a photosensitiser for acellular tumour structures, the abundance of these structures and their composition in the tumour is an important determinant. The importance of cellular tissue composition can be illustrated by the example of the spleen, which is known to contain considerably higher tissue levels of Photofrin and many other photosensitisers than a solid tumour (Bellnier *et al.*, 1989). Yet, we have found that Photofrin levels per individual spleen cell of a C3H mouse are several times lower than the average level of this photosensitiser in cells of a tumour growing in the same animal (M. Korbelik & G. Krosl, manuscript in preparation). This can be explained by the fact that the spleen contains many more cells per gram of tissue than the tumour.

Using enzymatic digestion, tumours and other tissues can be disaggregated into single-cell suspensions, and this allows the determination of photosensitiser levels per cell. However, the information on the average photosensitiser cellular level will probably still be inadequate to predict the direct killing of tumour cells by PDT. This becomes evident upon the examination of tumour cell suspensions, which reveals considerable heterogeneity in the cellular photosensitiser content (Korbelik, 1993). One of the main factors identified to be responsible for this heterogeneity is the presence of host immune cell populations infiltrating the tumour. Macrophages are the most prominent population of host cell infiltrate in many tumours. These cells retain markedly higher levels of Photofrin and other photosensitisers than the parenchymal malignant cells (Korbelik *et al.*, 1991; Korbelik, 1993; Korbelik & Krosl, 1993).

In our preliminary report (Korbelik & Krosl, 1993), we identified another factor that may be responsible for the heterogeneity of photosensitiser cellular content even within the malignant cell population. This factor is the non-uniformity of photosensitiser accumulation in cells located at different distances from the blood supply, which is examined in detail in the present report.

In order to address this issue, we have adapted a flow cytometry technique that was originally developed for studying the radiosensitivity of tumour cells relative to their blood vessel proximity (Olive *et al.*, 1985; Chaplin *et al.*, 1987). This technique is based on using the bisbenzimide DNA-binding fluorescent stain Hoechst 33342 (Hoechst). When administered intravenously, Hoechst clears rapidly from the circulation and, because of its diffusion/binding properties, its accumulation in tumour cells varies with their distance from the closest blood vessel (Durand *et al.*, 1990). We have used a dual laser flow cytometry instrument for simultaneous excitation of Hoechst and the photosensitiser, and for measurement of their fluorescence in single tumour cells.

### Materials and methods

### *Photosensitisers*

Photofrin and BPD (benzoporphyrin derivative monoacid) were obtained from QuadraLogic Technologies Phototherapeutics (Vancouver, BC, Canada). Bacteriochlorophyll-a, di- and tetrasulphonated tetraphenylporphines ($TPPS_2$ and $TPPS_4$) and di- and tetrasulphonated aluminium phthalocyanines ($AlPcS_2$ and $AlPcS_4$) were purchased from Porphyrin Products (Logan, UT, USA), while 5-aminolaevulinic acid (ALA) is produced by Sigma (St Louis, MO, USA). Photofrin was used as originally prepared in the physiological saline. Phosphate-buffered saline (PBS) was used to dissolve $AlPcS_2$, $AlPcS_4$, $TPPS_4$ and ALA. The stock solutions of BPD and $TPPS_2$ were prepared in dimethylsulphoxide and diluted 16 times in PBS immediately before administration. The stock solution of bacteriochlorophyll-a was first prepared in Hanks' balanced salt solution (HBSS) containing 10% Tween 80 (Sigma) and then further diluted in HBSS as described by Henderson *et al.* (1991).

### *Tumour models and PDT*

The tumours, SCCVII (squamous cell carcinoma) and FsaR fibrosarcoma, were grown in female C3H/HeN mice. Their

Correspondence: M. Korbelik.
Received 24 March 1994; and in revised form 3 June 1994

maintenance by biweekly transplantation and the implantation for the experiments (subcutaneous on the sacral region on the back of mice) was described previously (Korbelik, 1993). These two tumour models were chosen as representatives for carcinomas and sarcomas. One of the potentially relevant parameters in which they differ is the cell size, i.e. the SCCVII cells are considerably larger. The vascular perfusion of SCCVII tumour has been well characterised using Hoechst 33342 and other fluorescent probes (e.g. Olive *et al.*, 1985; Chaplin *et al.*, 1987). The tumours used in the experiments measured 200–300 mg wet weight, except where noted differently.

A tunable light source with a 1 kW xenon bulb (model A5000, Photon Technology International) was used for PDT. The tumours were treated by external irradiation with light delivered through a liquid light guide (5 mm core diameter, Oriel Corp.). The light was delivered with an output power of 41 mW (at 630 ± 10 nm) for Photofrin, 32 mW (at 674 ± 10 nm) for $AlPcS_2$ and 31 mW (at 690 ± 10 nm) for BPD. The fluence rate was 45 mW $cm^{-2}$ for Photofrin and 35 mW $cm^{-2}$ for $AlPcS_2$ and BPD. All tumours received a dose of 80 J $cm^{-2}$, which is in the curative range under the conditions of this study. The light treatment was performed 24 h after the administration of Photofrin or $AlPcS_2$, or 3 h after the administration of BPD. All hair in the area of the light treatment was removed by double shaving at the time of tumour implantation. The fluorescent probe Hoechst (Sigma) dissolved in PBS was administered intravenously via the lateral tail vein at 37.5 mg $kg^{-1}$ 30 min before the light treatment.

### *Flow cytometry and sorting*

The tumours growing in mice that were administered different photosensitisers and received Hoechst (37.5 mg $kg^{-1}$, i.v.) were excised 30 min after the Hoechst injection. After mincing, the tumours were dissociated into single-cell suspensions using an enzymatic digestion procedure described previously (Korbelik, 1993). The cells were resuspended in HBSS supplemented with 2% fetal bovine serum (FBS, HyClone Laboratories, Logan, UT, USA) and then analysed by flow cytometry using a dual-laser instrument FASC 440 (Becton Dickinson). Hoechst was excited by the UV laser and its fluorescence was detected through a 449 ± 5 nm bandpass filter. The 514 nm laser was employed for the excitation of photosensitisers and the emission of their fluorescence over 615 nm was recorded using a longpass filter. The exception were the experiments with $AlPcS_2$ and $AlPcS_4$ in which the photosensitiser was also excited by the UV laser (Korbelik, 1993).

The flow cytometry analysis of tumour cell suspensions based on Hoechst fluorescence was performed using a technique developed earlier in this laboratory with the SCCVII tumour model (Olive *et al.*, 1985). Briefly, the fluorescence intensity of Hoechst-stained cells was divided by the forward light scatter signal for each cell to obtain a measure of cellular concentration of the dye. The light scatter signals were also used to exclude cellular debris from the analysis. Tumour cells were grouped into ten fractions (sort windows) based on Hoechst concentration. Fraction 10 contained the 10% of cells with the highest value for Hoechst fluorescence intensity divided by cell size, i.e. the cells closest to the blood supply. Fraction 9 consisted of the 10% of cells with the next highest Hoechst concentration, etc. Fraction 1 represented the 10% of cells with the lowest Hoechst concentration, i.e. the cells most distant from the blood supply. In this study, the software program generated the average fluorescence readouts >610 nm per cell for each of the ten fractions.

In some experiments, the FsaR tumour cell suspensions were also stained with fluorescein isothiocyanate-conjugated goat anti-mouse IgG (Sigma) before flow cytometry in order to distinguish the FcR-positive cells (predominantly macrophages) and FcR-negative cells (mostly malignant cells). As described previously (Korbelik *et al.*, 1991), the antibody was added to the cell suspension for a brief (2 min) incubation at 37°C. The cells were then washed by centrifugation and kept on ice until the flow cytometry analysis. Fluorescein was excited by the 488 nm laser and the emission detected using a 530 ± 15 nm bandpass filter. In these experiments, the UV laser was used to excite both Hoechst and Photofrin.

The data reported are based on the analysis of $10^4$ cells in each sample, and expressed as the average value per cell obtained with five or more tumours (except when representative examples of individual tumours are shown). The values for photosensitiser fluorescence per cell were given after subtracting the average per cell background red fluorescence for the individual Hoechst fractions obtained with the tumours excised from mice not injected with a photosensitiser.

The same flow cytometry technique was used with single-cell suspensions from the PDT-treated tumours, except that a known number of cells from Hoechst fractions 1, 4, 7, 9 and 10 was sorted under sterile conditions into the tubes filled with 4 ml of α medium (Gibco, Burlington, Ontario, Canada) supplemented with 10% FBS. The content of the tubes was transferred into 60 mm plastic Petri dishes (Falcon 3002, Becton Dickinson) and the tubes were rinsed twice with 1 ml of the cell growth medium to ascertain that all the cells are transferred into the Petri dish. The samples in Petri dishes were left in a carbon dioxide incubator (37°C) in the dark for 10 days to allow colony formation. The colonies were then stained and counted to calculate the cell survival. The data presented are based on colony count in the three replicate Petri dishes (five replicates for the controls) and give average values for six identically treated tumours.

### Results

Hoechst administered intravenously to tumour-bearing mice exhibits a wide range of retention levels in the tumour cells. Studies published by other investigators (Olive *et al.*, 1985; Chaplin *et al.*, 1987) have suggested that *in situ* uptake of Hoechst in the SCCVII tumour depends primarily on cell location relative to the blood supply. The example of Hoechst distribution in SCCVII tumour cells obtained by flow cytometry analysis is shown in Figure 1a. The cells were divided into ten fractions according to the Hoechst concentration (fluorescence intensity corrected for the cell size) each containing 10% of the total population. The cells in fraction 10 showed the highest Hoechst levels, which implies that they were the closest to the blood supply, while fraction 1 contained the cells with the lowest Hoechst levels, i.e. those that were the most distant from the vasculature.

The same type of analysis was performed with another tumour model, the FsaR fibrosarcoma, with results very similar to those obtained with the SCCVII tumour, as shown also in Figure 1a. With both tumours, there was more than a 10-fold difference in Hoechst accumulation between fractions 1 and 10.

An example of the application of using Hoechst as the tumour perfusion probe for the analysis of the distribution of photosensitisers in tumour cells located at different distances from the blood supply is shown in Figure 1b. The photosensitiser examined was $TPPS_4$, which was administered intravenously into the SCCVII tumour-bearing mice 24 h before tumour excision. As in all the other experiments in this study, mice received Hoechst (37.5 mg $kg^{-1}$, i.v.) 30 min before the tumour excision. The distribution of $TPPS_4$ in the ten Hoechst fractions is shown for five individual tumours. For comparison, a curve for a tumour not containing $TPPS_4$ is also shown. It can be seen that $TPPS_4$ levels in tumour cells decrease with the increased distance of these cells from the nearest blood vessel.

The analysis of $TPPS_4$ distribution in tumour cells relative to their proximity to the blood supply is also shown in Figure 2. The data in this figure are based on the average of values obtained with a group of identically treated tumours corrected for the background red fluorescence of tumour cells. A possible influence of two different routes of photosensitiser administration is also examined. No sig-

**Figure 1** Representative examples of distribution of Hoechst 33342 concentration in cells of SCCVII and FsaR tumour and the profiles of cellular levels of $TPPS_4$ in individual tumours in relation to Hoechst tumour perfusion. **a**, Tumour-bearing mice received Hoechst (37.5 mg $kg^{-1}$, i.v.) 30 min before the tumours were excised and single-cell suspensions were rendered for flow cytometry analysis. Based on Hoechst concentration (fluorescence intensity in arbitrary units, a.u., divided by forward light scatter signal serving as a measure for cell size) tumour cells were grouped into ten equal fractions indicative of cell location relative to the blood supply. **b**, In addition to Hoechst, the mice bearing SCCVII tumour received $TPPS_4$ (25 mg $kg^{-1}$, i.v.) 24 h before the tumour excision. The photosensitiser fluorescence in the ten Hoechst fractions is shown for five individual tumours (curves 2–6). Also shown is the red fluorescence in the cells from a tumour excised from a mouse not injected with $TPPS_4$ (curve 1).

**Figure 2** The distribution of $TPPS_4$ in SCCVII tumour cells relative to their proximity to the blood supply. The photosensitiser was administered either intravenously (▼) or intraperitoneally (▲) (25 mg $kg^{-1}$) 24 h before the tumour excision. Hoechst was administered and the flow cytometry analysis based on the ten Hoechst fractions was performed as described in Figure 1. The data represent the average from five tumours. The bars show ± s.d.

nificant difference between intravenous and intraperitoneal administration can be detected; in both cases the $TPPS_4$ content in tumour cells decreased constantly with the cell distance from the blood supply, dropping in fraction 1 to 35–40% of the values measured in fraction 10.

The same type of analysis was applied with other photosensitisers in this study. The results with di- and tetrasulphonated derivatives of tetraphenylporphyine and aluminium phthalocyanine are shown in Figure 3. The data in this figure are presented as values relative to the photosensitiser level in the cells nearest to the tumour blood vessel (fraction 10). In arbitrary units of photosensitiser fluorescence, the values for fraction 10 obtained with disulphonated and tetrasulphonated forms of tetraphenylporphine were not statistically different (not shown). Di- and tetrasulphonated aluminium phthalocyanines also showed very similar cellular levels. The data in Figure 3 suggest that there is no significant difference in cellular distribution depending on the tumour blood supply between disulphonated and tetrasulphonated forms of either tetraphenylporphine or aluminium phthalocyanine. However, the aluminium phthalocyanines showed better diffusion to cells more distant from the blood supply than the tetraphenylporphines. A notable decrease in $AlPcS_2$ and $AlPcS_4$ levels can be observed only in the cells most remote from the vasculature.

The next set of experiments was designed to examine the factor of time elapsed after the photosensitiser administration. The first example (Figure 4a) shows the results with a photosensitiser characterised by relatively slow accumulation and long retention in tumours. It can be seen that higher levels of $AlPcS_2$ are accumulated in the tumour cells at 24 h than at 2 h after the photosensitiser administration. However, the diffusion profiles of $AlPcS_2$ for these two post-administration time intervals look very alike. Similar results were obtained with BPD, a photosensitiser that exhibits much faster tumour clearance. Its levels in tumour cells were markedly higher at 4 h than at 24 h post administration, but the pattern of BPD distribution between the ten Hoechst fractions was again very similar (Figure 4b). It can also be seen that BPD shows a very good accumulation in the tumour cells farthest from the blood supply.

The photosensitisers shown in Figure 5 are examples of photosensitiser fluorescence that comes from a metabolite of the drug administered to the tumour-bearing animals. The distribution of protoporphyrin IX, formed from the administered ALA (Kennedy *et al.*, 1990), was relatively uniform throughout fractions 10 to 3, with a decrease in the cellular levels seen only in the two cellular fractions most remote from the blood supply (Figure 5a). The results were quite different with bacteriopheophytin-a, the metabolite formed rapidly after the administration of bacteriochloro-

**Figure 3** The distribution of di- and tetrasulphonated forms of tetraphenylporphine and aluminum phthalocyanine in SCCVII tumour cells relative to their proximity to the blood supply. **a**, Mice administered $TPPS_2$ (◇) or $TPPS_4$ (△) (25 mg $kg^{-1}$, i.p.) 24 h before tumour excision. **b**, Mice given $AlPcS_2$ (◇) or $AlPcS_4$ (△) (10 mg $kg^{-1}$, i.v.) 24 h before excision. Hoechst was administered and flow cytometry analysis was performed as described in Figure 1. The data are given as values of the ratio to the photosensitiser fluorescence in the Hoechst fraction 10. The bars show ± s.d.

**Figure 4** The distribution of $AlPcS_2$ and BPD in SCCVII tumour cells relative to their proximity to the blood supply at different times after the photosensitiser administration. **a**, $AlPcS_2$ administered at 10 mg $kg^{-1}$ i.v. either at 2 (○) or 24 h (◇) before tumour excision. **b**, BPD administered at 5 mg $kg^{-1}$, i.v. either at 4 (○) or 24 h (□) before tumour excision. Hoechst was administered as described in Figure 1. The presentation of the data based on flow cytometry analysis is the same as in Figure 2. The bars show ± s.e.

phyll-a (Henderson *et al.*, 1991). Its levels decreased markedly in fraction 9, while very little, if any, photosensitiser was detectable in the cells most distant from the vasculature (Figure 5b).

The analysis of Photofrin distribution in tumour cells relative to the blood supply is shown in Figures 6 and 7. The comparison of the results obtained with the two different tumour types (Figure 6) reveals no significant differences. In both SCCVII and FsaR tumours, Photofrin exhibited relatively poor accumulation in cells removed from the blood supply. The level of this photosensitiser in the cells most distant from the vasculature was reduced to approximately one-fifth of the level detected in the cell fraction nearest to the blood vessel. These data, as well as those shown in the previous figures, are obtained with the tumours which ranged between 200 and 300 mg wet weight. A possible role of tumour size was also explored. The insert to Figure 6 shows the representative examples for a 'large' tumour (241 mg wet weight) and a 'small' tumour (19 mg wet weight). The smaller tumour exhibited better Photofrin accumulation than the larger tumour, in accordance with findings reported by other investigators (Bellnier *et al.*, 1989). However, the distribution of Photofrin in tumour cells relative to their proximity to the blood supply was not significantly different between these two tumours. This may be a reflection of a similar vascular perfusion of tumours within the size range examined.

Since tumour-associated macrophages (TAMs) have been shown to accumulate more Photofrin than the malignant tumour cells (Korbelik *et al.*, 1991; Korbelik, 1993), it was important to examine the photosensitiser diffusion profiles separately in these two different tumour cell populations. Using a technique described previously (Korbelik, 1993), the FsaR tumour cell suspension was separated into the Fc receptor-positive cells (predominantly TAMs) and the cells stained negatively for the Fc receptor (predominantly malignant cells) (Figure 7). Although, as expected, the $FcR^+$ population was characterised by several times higher Photofrin levels than the $FcR^-$ population, the photosensitiser distribution in cells relative to their proximity to the blood supply was, with both these populations, very similar to that depicted in Figure 6. The inset to Figure 7 shows that the content of the $FcR^+$ population in the FsaR tumour remains constant irrespective of the blood supply proximity; the same was reported previously for the SCCVII tumour (Olive, 1989).

The final experiments in this study were focused on the investigation of survival, following PDT *in vivo*, of tumour cells located at different distances from the blood supply. In addition to the administration of the photosensitiser and Hoechst, the tumours were treated with a light dose of

**Figure 5** The distribution of protoporphyrin IX and bacteriopheophytin-a, formed from the administered ALA and bacteriochlorophyll-a, respectively, in SCCVII tumour cells relative to their proximity to the blood supply. **a**, ALA (200 mg $kg^{-1}$, i.v.) administered 2 h before the tumour excision. **b**, Bacteriochlorophyll-a (32 mg $kg^{-1}$, i.p.) administered 24 h before the tumour excision. Hoechst was administered as described in Figure 1. Flow cytometry analysis data presentation as in Figure 2. The bars show ± s.d.

**Figure 6** The distribution of Photofrin in cells of SCCVII (▽) and FsaR (◇) tumours relative to their proximity to the blood supply. Photofrin (25 mg $kg^{-1}$, i.v.) was administered 24 h before tumour excision. The data given in the main graph were obtained with tumours measuring 200–300 mg wet weight (as was the case in the previous figures). The insert shows the representative examples of two FsaR tumours of different size. The wet weight measured 19 mg and 241 mg for the 'small' and 'large' tumour respectively. Hoechst administration and flow cytometry analysis was as in the previous figures. The bars show ± s.d.

Figure 7 The distribution of Photofrin in FcR-positive and FcR-negative cells of FsaR tumour relative to their proximity to the blood supply. The administration of Photofrin and Hoechst was as described in Figures 6 and 1 respectively. Before flow cytometry, the cells were stained for the Fc receptor as described in the Materials and methods section. The insert shows the content of FcR-positive cells in individual Hoechst fractions. The bars are ± s.e. (main graph) or ± s.d. (insert).

Figure 8 The survival of SCCVII tumour cells following PDT *in vivo* depending on their proximity to the blood supply. Hoechst (37.5 mg $kg^{-1}$, i.v.) was administered 30 min before the light treatment (80 J $cm^{-2}$). The tumours were excised immediately after the termination of the light treatment. Known numbers of tumour cells from Hoechst fractions 1, 4, 7, 9 and 10 and unsorted cells were plated for the colony formation assay. **a**, The survival of cells following Photofrin-based PDT (∇) (the photosensitiser administered as described in Figure 6), with the data for the light treatment only (Δ) (no Photofrin) also shown. **b**, The survival of cells following $AlPcS_2$- (∇) or BPD (Δ)-based PDT. The administration of $AlPcS_2$ (10 mg $kg^{-1}$, i.v.) was at 24 h before the light treatment, while BPD (2.5 mg $kg^{-1}$, i.v.) was given to mice 3 h before the light treatment. The bars are s.d.

80 J $cm^{-2}$ and they were excised immediately after the termination of the light treatment. The object of excising immediately after PDT was to assess the direct killing effect on tumour cells without the complicating influence of vascular-mediated cell killing, which has an increasingly greater contribution with the time the tumour is left *in situ*. The cells from different Hoechst fractions were sorted by flow cytometry and plated for the colony formation. The results with Photofrin are shown in Figure 8a. The survival of cells located closer to the tumour blood supply (fractions 10, 9 and 7) was 3–4 times lower than the survival of cells that are most remote from the vasculature. It should be noted that the survival data were normalised for the plating efficiency (PE) of control cells for each of the fractions obtained from the non-treated tumour. The PEs ranged typically between 15 and 30%; within the same tumour the highest PE were in fraction 7, and those for fractions 10 and 1 were lower by 5–10%. The data for the treatment with light only (no Photofrin), which are also included in Figure 8a, confirm that in this case there was no effect on cell survival. The colony formation of cells from the tumours with Photofrin not exposed to the light treatment was also not different from the controls (not shown).

The same type of experiment was performed with two other photosensitisers (Figure 8b). With $AlPcS_2$, which is known to be more effective than Photofrin in direct tumour cell killing by PDT (Henderson & Farrell, 1989), the tumour cells nearest to the vasculature also showed the lowest survival. Tumour cell killing decreased steadily with the increase in the distance of the cells from the blood supply, showing an aproximately 6-fold difference in survival between the cells of fractions 1 and 10. In contrast, the results with BPD indicate only a very limited decrease in the survival of tumour cells following PDT. The survival level of tumour cells ranged around 80% and showed no change in relation to their proximity to the blood supply. In all the experiments presented in Figure 8 the cell yield per gram of tumour tissue was the same as the control. The PDT treatments of SCCVII tumour shown in Figure 8 (including those with BPD) were equieffective in tumour control, resulting in 15–20% tumour cures (not shown).

### Discussion

The results presented demonstrate that there are substantial differences among photosensitisers in their potential to reach tumour cells distant from the vasculature. Among the photosensitisers tested, the best properties in this respect were exhibited by BPD, which showed no decrease in the cellular content even in the tumour cells most remote from the blood supply. This is the attribute that would be desirable for achieving a high level of direct killing of tumour cells by PDT. With respect to this quality, the other photosensitisers examined followed BPD in this decreasing order: ALA > $AlPcS_2$/$AlPcS_4$ > $TPPS_2$/$TPPS_4$ > Photofrin > bacteriochlorophyll-a. The uniformity of BPD distribution among the ten Hoechst fractions presumably reflects good penetration of this drug from the circulation into the cells that are most distant from the vasculature. The diffusion potential seen with different photosensitisers was not paralleled by the degree of their lipophilicity. This also implies that the affinity of photosensitisers for binding to specific classes of plasma proteins (lipoproteins or albumin) (Kessel, 1986) is not a decisive factor. The results with di- and tetrasulphonated forms of aluminium phthalocyanine and tetraphenylporphine showing no significant differences in the distribution pattern between the ten Hoechst fractions support this suggestion.

It should be noted that $AlPcS_4$ and $TPPS_4$ have been reported to localise preferentially in the acellular tumour structures, while $AlPcS_2$ and $TPPS_2$ are retained more selectively in tumour cells (Peng *et al.*, 1990). The observation in this study that there is very little, if any, difference in cellular accumulation between di- and tetrasulphonated forms of these photosensitisers is not contradictory to this finding; the levels of $AlPcS_4$ and $TPPS_4$ in the acellular tumour structures were not measured in this work and they may be much higher than those in the tumour cellular compartment.

The data shown in this work further indicate that the photosensitiser property to reach tumour cells removed from the vasculature is not dependent on tumour type (SCCVII *vs* FsaR tumour, Figure 6), tumour size (Figure 6), the route of the administration (i.p. *vs* i.v., Figure 2), the time after administration (Figure 4) or on the characteristics of host cell infiltrate in the tumour (Figure 7). This leaves the possibility that a certain property of the photosensitiser molecule not identified at present is relevant in this respect. The data with ALA may suggest that this small molecule penetrates well into regions removed from the vasculature, but the cells that are the most distant from the blood supply may be too compromised metabolically to efficiently synthesise protoporphyrin IX.

The examination of the survival of SCCVII tumour cells located at different distances from the blood supply following the PDT treatment *in vivo* demonstrates that the direct killing effect of PDT depends on the blood vessel proximity of target cells. The examples with Photofrin- and $AlPcS_2$-based PDT (Figure 8) show that the greatest killing effect was inflicted on the tumour cells nearest to the vasculature, while cell survival increased with increased distance from the nearest blood vessel. Such a response to PDT may reflect variations in tumour cellular levels of both photosensitiser and oxygen. Earlier studies with several mouse tumour models have demonstrated the existence of radiobiological anoxia in Hoechst fractions most distant from the blood supply (Chaplin *et al.*, 1985, 1987). However, the decrease in tumour cell killing with $AlPcS_2$-based PDT was already evident in the fractions relatively close to the vasculature which have not shown a decline in the photosensitiser level and are well oxygenated in the non-treated tumour. This may indicate that a reduction in the oxygen supply was induced in these cells during the photodynamic light treatment, a phenomenon suggested by Henderson and Fingar (1989). Strategies of adjuvant treatments to be combined with PDT, which would improve tumour oxygenation during the light irradiation, should allow a greater killing effect at least in these intermediate regions that exhibit sufficiently high cellular levels of $AlPcS_2$.

The relevance of photosensitiser diffusion potential, once it has penetrated the tumour vascular lining, is thus clearly established in this study. The example with BPD, which shows an optimal diffusion quality but exhibits a poor direct cytotoxic effect, demonstrates that both these properties are prerequisites for an ideal photosensitiser. However, the excellent diffusion property of BPD may still contribute to the efficacy of this photosensitiser in tumour eradication based on the indirect effects of PDT.

Expert technical assistance was provided by Sandy Lynde (tumour cell preparations) as well as by Denise McDougal and Nancy LePard (flow cytometry). The authors thank Dr Ralph Durand for advice in flow cytometry analysis. The study was supported by Grant MA-12165 from the Medical Research Council of Canada.

### References

BELLNIER, D.A., HO, Y.-K., PANDEY, R.K., MISSERT, J.R. & DOUGHERTY, T.J. (1989). Distribution and elimination of Photofrin II in mice. *Photochem. Photobiol.*, **50**, 221-228.

CHAPLIN, D.J., DURAND, R.E. & OLIVE, P.L. (1985). Cell selection from a murine tumour using the fluorescent probe Hoechst 33342. *Br. J. Cancer*, **51**, 569-572.

CHAPLIN, D.J., OLIVE, P.L. & DURAND, R.E. (1987). Intermittent blood flow in a murine tumor: radiobiological effects. *Cancer Res.*, **47**, 597-601.

DURAND, R.E., CHAPLIN, D.J. & OLIVE, P.L. (1990). Cell sorting with Hoechst or carbocyanine dyes as perfusion probes in spheroids and murine tumors. In *Methods in Cell Biology*, Darzynkiewicz, Z. & Crissman, H.A. (eds) pp. 509-518. Academic Press: New York.

HENDERSON, B.W. & FARRELL, G. (1989). Possible implications of vascular damage for tumor cell inactivation *in vivo*: comparison of different photosensitizers. *SPIE*, **1065**, 2-10.

HENDERSON, B.W. & FINGAR, V.H. (1989). Oxygen limitation of direct tumor cell kill during photodynamic treatment of a murine tumor model. *Photochem. Photobiol.*, **49**, 299-304.

HENDERSON, B.W., SUMLIN, A.B., OCWCZARCZAK, B.L. & DOUGHERTY, T.J. (199i). Bacteriochlorophyll-a as photosensitiser for photodynamic treatment of transplantable murine tumors. *J. Photochem. Photobiol., B: Biol.*, **10**, 303-313.

KENNEDY, J.C., POTTIER, R.H. & PROSS, D.C. (1990). Photodynamic therapy with endogenous protoporphyrin IX: basic principles and present clinical experience. *J. Photochem. Photobiol., B: Biol.*, **6**, 143-148.

KESSEL, D. (1986). Porphyrin-lipoprotein association as a factor in porphyrin localization. *Cancer Lett.*, **33**, 183-188.

KORBELIK, M. (1993). Distribution of disulfonated and tetrasulfonated aluminum phthalocyanine between malignant and host cell populations of a murine fibrosarcoma. *J. Photochem. Photobiol., B: Biol.*, **20**, 173-181.

KORBELIK, M. & KROSL, G. (1993). Distribution of photosensitisers between tumor cells and tumor infiltrating host cells. *SPIE*, **2078**, 389-396.

KORBELIK, M., KROSL, G., OLIVE, P.L. & CHAPLIN, D.J. (1991). Distribution of Photofrin between tumour cells and tumour associated macrophages. *Br. J. Cancer*, **64**, 508-512.

OLIVE, P.L. (1989). Distribution, oxygenation, and clonogenicity of macrophages in a murine tumor. *Cancer Commun.*, **1**, 93-100.

OLIVE, P.L., CHAPLIN, D.J. & DURAND, R.E. (1985). Pharmacokinetics, binding and distribution of Hoechst 33342 in spheroids and murine tumours. *Br. J. Cancer*, **52**, 739-746.

PASS, H.I. (1993). Photodynamic therapy in oncology: mechanisms and clinical use. *J. Natl Cancer Inst.*, **85**, 443-456.

PENG, Q., MOAN, J., FARRANTS, G., DANIELSEN, H.E. & RIMINGTON, C. (1990). Localization of potent photosensitizers in human tumor LOX by means of laser scanning microscopy. *Cancer Lett.*, **53**, 129-139.